# Histamine induces microglia activation and dopaminergic neuronal toxicity via H1 receptor activation

**DOI:** 10.1186/s12974-016-0600-0

**Published:** 2016-06-04

**Authors:** Sandra M. Rocha, Tatiana Saraiva, Ana C. Cristóvão, Raquel Ferreira, Tiago Santos, Marta Esteves, Cláudia Saraiva, Goun Je, Luísa Cortes, Jorge Valero, Gilberto Alves, Alexander Klibanov, Yoon-Seong Kim, Liliana Bernardino

**Affiliations:** Health Sciences Research Centre, Faculty of Health Sciences, University of Beira Interior, Covilhã, Portugal; Burnett School of Biomedical Sciences, College of Medicine, University of Central Florida, Orlando, FL USA; Center for Neuroscience and Cell Biology, Coimbra, Portugal; Division of Cardiovascular Medicine and Department of Biomedical Engineering, University of Virginia, Charlottesville, VA USA; Health Sciences Research Centre, University of Beira Interior, Av. Infante D. Henrique, 6200-506 Covilhã, Portugal

**Keywords:** Histamine, Microglia, Phagocytosis, NADPH oxidase, Neurotoxicity, Dopaminergic neurons

## Abstract

**Background:**

Histamine is an amine widely known as a peripheral inflammatory mediator and as a neurotransmitter in the central nervous system. Recently, it has been suggested that histamine acts as an innate modulator of microglial activity. Herein, we aimed to disclose the role of histamine in microglial phagocytic activity and reactive oxygen species (ROS) production and to explore the consequences of histamine-induced neuroinflammation in dopaminergic (DA) neuronal survival.

**Methods:**

The effect of histamine on phagocytosis was assessed both in vitro by using a murine N9 microglial cell line and primary microglial cell cultures and in vivo. Cells were exposed to IgG-opsonized latex beads or phosphatidylserine (PS) liposomes to evaluate Fcγ or PS receptor-mediated microglial phagocytosis, respectively. ROS production and protein levels of NADPH oxidases and Rac1 were assessed as a measure of oxidative stress. DA neuronal survival was evaluated in vivo by counting the number of tyrosine hydroxylase-positive neurons in the substantia nigra (SN) of mice.

**Results:**

We found that histamine triggers microglial phagocytosis via histamine receptor 1 (H1R) activation and ROS production via H1R and H4R activation. By using apocynin, a broad NADPH oxidase (Nox) inhibitor, and Nox1 knockout mice, we found that the Nox1 signaling pathway is involved in both phagocytosis and ROS production induced by histamine in vitro. Interestingly, both apocynin and annexin V (used as inhibitor of PS-induced phagocytosis) fully abolished the DA neurotoxicity induced by the injection of histamine in the SN of adult mice in vivo. Blockade of H1R protected against histamine-induced Nox1 expression and death of DA neurons in vivo.

**Conclusions:**

Overall, our results highlight the relevance of histamine in the modulation of microglial activity that ultimately may interfere with neuronal survival in the context of Parkinson’s disease (PD) and, eventually, other neurodegenerative diseases which are accompanied by microglia-induced neuroinflammation. Importantly, our results also open promising new perspectives for the therapeutic use of H1R antagonists to treat or ameliorate neurodegenerative processes.

**Electronic supplementary material:**

The online version of this article (doi:10.1186/s12974-016-0600-0) contains supplementary material, which is available to authorized users.

## Background

Histamine is a biogenic amine with extensive effects on several peripheral immune cells and recently highlighted as a promising modulator of brain innate immunity. In the brain, it is produced by histaminergic neurons, mast, and microglial cells [1–6]. Histamine exerts its various functions through the activation of four distinct subtypes of G protein-coupled receptors (H1R, H2R, H3R, and H4R) [7, 8]. Microglial cells, the innate immune cells in the central nervous system, express all histamine receptors [9], and we were the first to show that histamine modulates N9 microglial cells migration and interleukin-1β (IL-1β) release [7]. Other studies also showed that histamine may also modulate the release of IL-1β, tumor necrosis factor (TNF)-alpha, IL-6, nitric oxide (NO), reactive oxygen species (ROS) production and induce mitochondrial membrane potential dysfunction [8–13].

Interestingly, the functional consequences of histamine in microglial phagocytosis remain unexplored. Microglia phagocytose foreign pathogens, stressed/apoptotic cells, and also supernumerary synapses in postnatal development. Several microglia receptors enable the recognition of these targets, namely Fc-gamma receptors (FcγR), which bind to IgG of opsonized particles, and phosphatidylserine (PS) receptors (PSR), which bind to PS on the outer membrane leaflet of stressed and apoptotic cells. Several other receptors can recognize the exposed PS, such as T cell/transmembrane, immunoglobulin, and mucin (TIM) -1, TIM-4, and the vitronectin receptor [14–16]. Overall, phagocytosis is considered to be beneficial since it eliminates stressed/apoptotic cells and maintains healthy neuronal networks. However, phagocytosis can also activate the respiratory burst cascade, which produces toxic ROS ultimately inducing cytotoxicity [16, 17]. In particular, the nicotinamide adenine dinucleotide phosphate (NADPH) oxidase (Nox) system is responsible for producing ROS that are associated with microglial phagocytosis and neuronal death [18, 19]. Accordingly, herein, we found that histamine induces microglial phagocytosis via H1R activation and ROS production via both H1R and H4R activation. Both microglial responses depend on the activation of the Nox system. Therefore, histamine may have an impact on the pathogenesis of brain diseases which are associated with inflammatory conditions. In fact, increased levels of histamine found in the cerebrospinal fluid (CSF) and in brain parenchyma as well as pharmacological experiments and clinical trials suggest that the histaminergic system may be implicated in the etiology and/or progression of several neurodegenerative diseases [20, 21] such as Parkinson’s disease (PD) [22–25]. In fact, it has been shown that the substantia nigra (SN) is a brain region highly vulnerable to the neurotoxic actions of microglia [14, 26] and in particular to histamine [27]. Recently, we also showed that the secretome of microglial cells exposed to histamine induces degeneration of dopaminergic (DA) neurons in vitro [8]. However, the cellular and molecular mechanisms behind these toxic effects are not known. Importantly, our present results also highlight the potential of H1R antagonists to prevent DA cell loss derived from histamine-induced microglial neuroinflammation in vivo. Altogether, we present novel findings that unravel the effects of histamine in microglial-induced DA neuronal death and the potential therapeutic application of H1R antagonist to counteract these toxic effects.

## Methods

### N9 microglial cell line cultures

Murine N9 microglia cell line was grown in modified Roswell Park Memorial Institute (RPMI) medium (Sigma-Aldrich, St. Louis, MO, USA) during 24 h at 37 °C in 5 % CO_2_ and 95 % atmospheric air, as previously described by us [28]. Cells were plated at a density of 2 × 10^4^ cells per well in 24-well trays (phagocytic studies and immunocytochemistry), 5 × 10^5^ cells per well in 6-well trays (protein extraction), or 5 × 10^4^ cells per well in 96-well trays (ROS measurements). Cell treatments included the following incubation setup: histamine dihydrochloride (1, 10, and 100 μM, Sigma-Aldrich), H1R antagonist (mepyramine maleate, 1 μM), H2R antagonist (cimetidine, 5 μM), H3R antagonist (carcinine ditrifluoroacetate, 5 μM), H4R antagonist (JNJ7777120, 5 μM), H1R agonist (2-pyridylethylamine dihydrochloride, 100 μM), H4R agonist (4-methylhistamine dihydrochloride, 20 μM) (all from Tocris Bioscience, Bristol, UK), and apocynin (5 μM, Sigma-Aldrich). All histamine receptor antagonists and apocynin were added 1 h prior to cell treatments and maintained during the course of the experiments.

### Primary murine microglia cell cultures

Postnatal whole brain microglia cultures of wild-type (WT) and Nox1 knockout (Nox1-KO) mice were prepared as previously reported [29]. Briefly, the whole brain of 3–5-day postnatal mice pups were dissected, carefully stripped of meninges, and mechanically dissociated with a 5-ml pipette, followed by 5–10 sequential passages through 20-, 22-, and 25-gauge needles. Finally, cells were passed through a 70 μm mesh, pelleted by centrifugation, suspended in high-glucose Dulbecco’s modified Eagle medium (DMEM) with 10 % fetal bovine serum (FBS) and 100 units/ml penicillin plus 100 μg/ml streptomycin (all from Sigma-Aldrich), and plated onto 25 cm^2^ poly-D-lysine-coated culture flasks (one brain per flask). The cultures were kept at 37 °C in a 5 % CO_2_, 95 % air atmosphere. The medium was changed every 4 days. On day 14, the culture flasks were shaken during 2 h at 200 rpm to detach microglial cells, leaving astrocytes in an adherent monolayer. The microglia-enriched supernatant was collected and centrifuged for 10 min at 1200 rpm. The pellet was then suspended in DMEM, and the microglial cells were plated onto poly-d-lysine-coated coverslips and kept at 37 °C in a 5 % CO2, 95 % air atmosphere until used. The culture medium was replaced every 4 days. Each pup was genotyped to confirm its phenotype as WT or Nox1-KO.

### In vitro phagocytosis assays

#### FcγR-mediated phagocytosis

Latex beads (Sigma-Aldrich) were opsonized with rabbit IgG (1 μg/ml, Sigma-Aldrich) under constant agitation overnight at 4 °C. Then, the beads were resuspended in modified RPMI medium without NaHCO_3_ (Sigma-Aldrich) and distributed at a density of 1 × 10^5^ beads per well, over cells that were previously exposed to histamine for 6 h. After 40 min of incubation with the beads, the cells were washed with phosphate-buffered saline (PBS) and fixed with 4 % paraformaldehyde (PFA, Sigma-Aldrich) for 20 min at room temperature (RT). Extracellular and/or adherent beads were labeled with secondary antibody Alexa Fluor 594 donkey anti-rabbit (1:500, Life Technologies Ltd, Paisley, UK) in PBS, for 1 h at RT. For nuclear labeling, cell preparations were stained with Hoechst 33342 (2 μg/ml, Life Technologies Ltd.) in PBS, for 5 min at RT. Coverslips were then mounted in Dako fluorescent medium (Dakocytomation Inc., Glostrup, Denmark). Confocal images were acquired using an Axio Observer LSM710 confocal microscope (Zeiss) under a 63× oil objective. For each coverslip, five photomicrographs were acquired in order to capture stained nuclei (in blue), extracellular and/or adherent beads (in red), and the total number of beads (differential interference contrast image). The location of each bead was analyzed by comparing the three separate images simultaneously. Only beads without fluorescent labeling were considered as internalized particles.

#### PS-mediated phagocytosis

Fluorescent-labeled PS or phosphatidylcholine (PC)-containing liposomes (5 μl/well) were added to the cells previously exposed to histamine for 6 h. PC liposomes were used as a negative control for PS-dependent phagocytosis. To block PS-induced phagocytosis, annexin V (4 μl/well), which binds to PS residues, was added 1 h prior to incubation with liposomes. After 2 h of exposure to liposomes, the cells were washed with RPMI medium and then fixed with 4 % PFA. After several rinses with PBS, unspecific binding was prevented by incubating cells in a 3 % BSA and 0.5 % Triton X-100 solution (all from Sigma-Aldrich) in PBS, for 30 min at RT. Then, the cells were incubated overnight at 4 °C with primary antibody rat monoclonal anti-CD11b (1:600; AbD Serotec, Oxford, UK) diluted in PBS containing 0.3 % BSA and 0.1 % Triton X-100. After several washes with PBS, the cells were incubated for 1 h at RT with the corresponding secondary antibody Alexa Fluor 488 goat anti-rat (1:200; Life Technologies Ltd) diluted in PBS. For nuclear labeling, cell preparations were stained with Hoechst 33342 (2 μg/ml; Life Technologies Ltd) in PBS, for 5 min at RT and mounted in a Dako fluorescent medium. Fluorescent images were acquired using an Axio Observer LSM710 confocal microscope (Zeiss) under a 40× oil objective. Photomicrographs were acquired using the same acquisition settings in each experimental protocol. For each experimental setting, we first outlined microglial cytoplasm by staining CD11b (in green) and the corrected fluorescence intensity of PS/PC liposomes (in red) was subtracted from the fluorescence background. Similar protocols reported by others were used to quantify microglial phagocytosis of synaptic inputs (presynaptic terminals), axonal or myelin debris, and neurofilament-positive axonal material [30–32]. Quantification of fluorescence intensity of the PS/PC liposomes was performed with at least 64 cells per condition. Analysis was performed using the ImageJ software.

### Liposome preparation

Liposomes (diameter 2–6 μm) were prepared by a classical Bangham technique. Multilamellar vesicles were chosen to maximize the fluorescent-dye load per particle without significant self-quenching [33]. Briefly, dioleoyl phosphatidylcholine (Avanti Polar Lipids, Alabama, USA) and cholesterol (Sigma-Aldrich) were mixed in chloroform at 1:1 molar ratio, and DiIC18(3) dye (Life Technologies Ltd.) was added; this dye has two stearyl residues attached to the fluorochrome, so it is tightly attached to the liposome membrane and is not exchangeable. PS (Avanti Polar Lipids) was added at 1:10 PS to PC mass ratio to the experimental sample but not to control. After chloroform removal by rotary evaporation and argon gas flush, vacuum desiccation was applied to remove traces of organic solvent. After the complete removal of the solvent, normal saline was added to the lipid film and the sample was subjected to hydration under vortexing.

### Measurements of cellular ROS levels

ROS levels were measured using a dihydroethidium probe (DHE, Life Technologies Ltd), as described previously by us [34]. In the DHE assay, blue fluorescent DHE is dehydrogenated by superoxide (O_2_^−^) to form red fluorescent ethidium bromide. The cells exposed for 2 h to stimuli were then incubated for 4 h (N9 cell line) or 20 min (primary murine microglial cells) with 100 μM DHE in culture medium at 37 °C. The emitted fluorescence (excitation 515 nm; emission 605 nm) was read in a spectrofluorometer (SpetroMax GeminiEM; Molecular Devices).

### Immunocytochemistry

The cells were fixed with 4 % PFA, and unspecific binding was prevented by incubating cells in a 3 % BSA and 0.5 % Triton X-100 solution (all from Sigma-Aldrich) for 30 min at RT. The cells were incubated with primary antibodies prepared in 0.3 % BSA and 0.1 % Triton X-100, kept overnight at 4 °C, washed with PBS, and finally incubated for 1 h at RT with the corresponding secondary antibody. Antibodies used were as follows: rat monoclonal anti-CD11b (1:600; AbD Serotec), mouse monoclonal anti-acetylated α-tubulin (1:100; Sigma-Aldrich), goat polyclonal anti-Nox1 (1:250; Santa Cruz Biotechnology, Heidelberg, Germany), Alexa Fluor 594 goat anti-rat, Alexa Fluor 488 goat anti-rat, Alexa Fluor 488 donkey anti-goat, and Alexa Fluor 594 rabbit anti-mouse (all 1:200 in PBS, from Life Technologies Ltd). Membrane ruffling was observed using phalloidin, a marker for filamentous actin. The cells were incubated for 2 h with Alexa Fluor 594 conjugated to phalloidin (1:100; Life Technologies Ltd) in PBS, at RT. For nuclear labeling, cell preparations were stained with Hoechst 33342 (2 μg/ml, Life Technologies Ltd) in PBS, for 5 min at RT and mounted in Dako fluorescent medium. Fluorescent images were acquired using an Axio Observer LSM710 confocal microscope (Zeiss) under a 63× oil objective.

### Western blotting

The cells were washed with ice-cold PBS and lysed on ice in RIPA buffer (50 mM Tris-HCl, pH 8.0, 150 mM NaCl, 2 mM sodium orthovanadate, 1 % Nonidet-P40, 0.5 % sodium deoxycholate, 0.1 % SDS, and 1 % of a protease inhibitor mixture containing 4-(2-aminoethyl)benzenesulfonyl fluoride hydrochloride, pepstatin A, E-64, bestatin, leupeptin, and aprotinin, all from Sigma-Aldrich). After gentle homogenization, the total amount of protein was quantified using the Bradford method (Bio-Rad Protein Assay, Bio-Rad, Hertz, UK). Afterwards, the samples were loaded onto 12 % acrylamide/bisacrilamide gels (Bio-Rad). Proteins were separated by SDS-PAGE and then transferred to PVDF membranes (Amersham Hybond^TM^-P, GE Healthcare, Little Chalfont, UK). The membranes were then blocked with 5 % non-fat milk in Tris-buffered saline with 0.1 % Tween 20 (TBS-T) for 1 h at RT. Incubation with mouse monoclonal anti-acetylated α-tubulin (1:200; Sigma-Aldrich), goat polyclonal anti-Nox1 (1:200; Santa Cruz Biotechnology), goat polyclonal anti-Nox2 (1:2000; BD Laboratories, San Jose, CA, USA), goat polyclonal anti-Nox4 (1:200; Santa Cruz Biotechnology), and mouse monoclonal anti-Rac1 (1:750; Millipore, Billerica, MA, USA) diluted in TBS-T was done overnight at 4 °C. After rinsing three times with TBS-T, the membranes were incubated for 1 h at RT with an anti-mouse (1:10,000; GE Healthcare) or anti-goat antibody (1:10,000; Santa Cruz Biotechnology) diluted in TBS-T. The membranes were then incubated with ECF substrate (ECF Western Blotting Reagent Packs, GE Healthcare) for 5 min. Protein bands were detected using the Molecular Imager FX system (Bio-Rad) and quantified by densitometry analysis using the Quantity One software (Bio-Rad).

### In vivo experiments

Wild-type C57BL/6 male mice (8 to 12 weeks) were maintained in appropriate cages, under temperature-controlled environment under a 12 h light/dark cycle with free access to food and water. All experiments related to the use of experimental animal models were conducted in compliance with protocols approved by the national ethical requirements for animal research, the European Convention for the Protection of Vertebrate Animals Used for Experimental and Other Scientific Purposes (European Union Directive number 2010/63/EU). Before stereotaxic surgery, the mice were anesthetized with an intraperitoneal injection (i.p.) of ketamine and xylazine (90 mg/kg and 10 mg/kg, respectively).

To analyze DA neuronal survival, the mice were unilaterally injected with 2 μl of sterile PBS or histamine (100 μM in PBS) in the right lateral SN (coordinates related to the bregma: AP = +3.0 mm; ML = −1.4 mm; DV = −4.4 mm; according to [35]) using a stereotaxic frame (Stoelting 51600, IL, USA). In another set of animals, 2 μl of sterile H1R antagonist (mepyramine maleate, 1 μM) or H4R antagonist (JNJ7777120, 5 μM) were also injected following histamine administration. Stereotaxic injections were made using a 10 μl Hamilton syringe (Hamilton Company) at a rate of 0.2 μl/min during 10 min. The needle was removed and the incision sutured. Apocynin (4 mg/kg, i.p.) or annexin V (5 μg/mouse, intravenously (i.v.)) were administrated 30 min before and also 24 h after stereotaxic injections, as described previously by others [33, 36]. Seven-day (for DA neuronal survival analysis) or 3-day (for co-localization between CD11b+/TH+/annexin V, Nox1+/CD11b+ or Nox1+/TH+) upon all injections, the mice were transcardially perfused with 0.9 % NaCl and fixed with 4 % PFA. The brains were removed and post-fixed in 4 % PFA overnight at 4 °C. After fixation, the brains were cryoprotected in 30 % sucrose (in 0.1 M PBS) at 4 °C until sinking, frozen in liquid nitrogen, and maintained at −80 °C. Before sectioning, the brains were embedded in an optimal cutting temperature medium (OCT) and cut into 35-μm coronal sections using a cryostat-microtome (Leica CM3050S, Leica Microsystems) at −20 °C. Slices corresponding to the SN of each animal were collected for immunohistochemical processing, as described previously by us [37].

For the in vivo phagocytosis assays, the mice were stereotaxically injected into the right SN with 1 μl of PS liposomes followed by histamine treatment (as described above). Perfusion was performed 18 h after the stereotaxic injections. The brains were removed, fixed in 4 % PFA, and cut as stated previously. Floating sections (35 μm) were then analyzed for phagocytic cells (CD11b+ cells with liposome detection).

### Fluorescent immunohistochemistry staining

SN slices were permeabilized with 1 % Triton X-100 in 0.1 M PBS, for 45 min at RT. Non-specific binding sites were blocked with 10 % FBS in PBS for 30 min at RT. In a group of slices, permeabilization was performed only after incubation with Alexa Fluor 488 annexin V conjugate. Then, the slices were incubated with the primary antibodies: rat monoclonal anti-CD11b (1:600; AbD Serotec), goat polyclonal anti-Nox1 (1:200; Santa Cruz Biotechnology), and mouse monoclonal anti-TH antibody (1:1000; BD Laboratories), diluted in 10 % FBS in PBS, overnight at 4 °C. After several washes with 0.1 % Triton X-100 (PBS-T), the slices were incubated with the respective secondary antibodies, Alexa Fluor 594 or 488 goat anti-rat, Alexa Fluor 647 anti-mouse, or Alexa Fluor 546 anti-goat (all 1:200; Life Technologies Ltd) diluted in PBS, for 2 h at RT. Following nuclei counterstaining with Hoechst 33342 (2 μg/ml; Life Technologies Ltd) in PBS for 5 min at RT, the sections were mounted in glass slides with a Dako mounting medium. Fluorescent images were acquired using an Axio Observer LSM710 confocal microscope (Zeiss) under a 63× oil objective. To quantify microglial phagocytosis of liposomes, three distinct brain slices containing the SN [37] were selected per mouse, and in each slice, three pictures were taken within a range of approximately 200 μm from the liposomes injection site. The volume of CD11b-positive cells containing PS liposomes was then quantified by orthogonal analysis as described previously [33].

### Immunohistochemistry for Tyrosine Hydroxylase (TH)

Free-floating immunohistochemistry was initiated by incubating SN sections in a 10 mM citrate solution (pH 6.0) at 80 °C [37]. After cooled to RT, the slices were placed in water for 5 min and then washed in PBS-T. The sections were blocked with 10 % FBS in PBS for 1 h at RT and washed twice with PBS-T. For the inhibition of endogenous peroxidase activity, the brain sections were incubated for 10 min in 3 % H_2_O_2_. This step was followed by two washes with PBS-T. Incubation with mouse monoclonal anti-TH antibody (1:1000; BD Laboratories) diluted in 5 % FBS in PBS was performed overnight at 4 °C. After washing with PBS-T, the slices were incubated for 1 h at RT with a biotinylated goat anti-mouse antibody (1:200; Vector Laboratories, Peterborough, UK) diluted in 1 % FBS. The sections were washed again and incubated with the avidin–biotin peroxidase complex reagent (ABC kit from Vector Laboratories) for 30 min at RT. Then, the sections were developed using 3,3′-diaminobenzidine (Sigma-Aldrich) in TBS with 30 % H_2_O_2_, mounted onto Superfrost precleaned coated slides, dehydrated through a graded ethanol series, cleared using xylene and coversliped with Entellan mounting medium (Merck, Kenilworth, NJ, USA). Quantitative analysis of DA neurons in SN was carried out by serial section analysis of the total number of TH-positive neurons throughout the rostro-caudal axis. The region corresponding to the SN was carefully delineated and the total number of TH-positive neurons in the full extent of this structure was counted per section in each hemisphere, as described previously by us [37].

### Data analysis

Data are expressed as percentage of values obtained in control conditions or as percentage of the total number of cells and are presented as mean ± SEM of at least three independent experiments. Statistical analysis was performed using paired or unpaired *t* test (whenever appropriate) or one-way ANOVA followed by Bonferroni’s multiple comparison test, as indicated in the figure legends. Values of *P* < 0.05 were considered significant. All statistical procedures were performed using GraphPad Prism 5 (GraphPad Software Inc.).

## Results

### Histamine promotes microglial phagocytosis

**Fig. 1:**
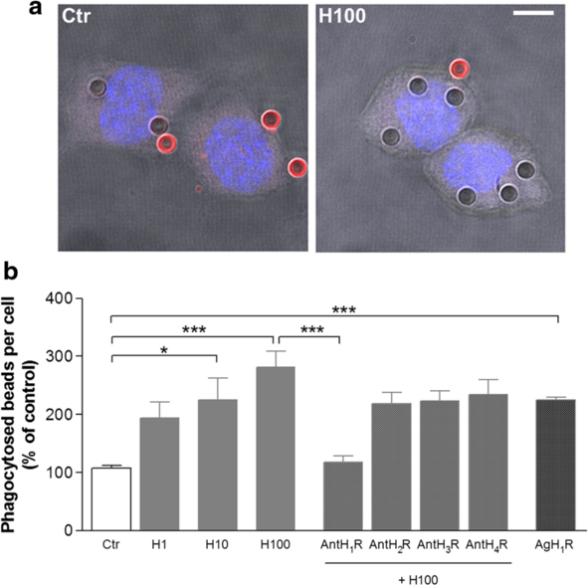
Histamine-induced microglial FcγR-mediated phagocytosis through H1R activation. **a** Representative photomicrographs illustrate the stimulatory effect of 100 μM histamine on microglial phagocytosis of IgG latex beads. Non-ingested beads display *red labeling* whereas ingested beads do not show any fluorescence signal. *Scale bar* 10 μm. **b** Only 10 (*H10*) and 100 (*H100*) μM histamine significantly increased the number of phagocytosed IgG latex beads per cell as compared with untreated controls (*Ctr*). Microglial phagocytosis induced by 100 μM histamine was fully blocked by an H1R antagonist (AntH1R, mepyramine maleate, 1 μM) and mimicked by an H1R agonist (AgH1R, 4-methylhistamine dihydrochloride, 20 μM). The involvement of other receptors was excluded since the application of their respective antagonists (“Ant”) did not interfere with the phagocytosis-inducing effect of histamine (H2R antagonist (cimetidine), 5 μM; H3R antagonist (carcinine ditrifluoroacetate), 5 μM; and H4R antagonist (JNJ7777120), 5 μM). Data are expressed as mean ± SEM (*n* = 5–15). *P < 0.05 and ***P < 0.001, using one-way ANOVA followed by Bonferroni’s multiple comparison test

We first evaluated the effect of histamine on FcγR-mediated phagocytosis in murine N9 microglial cell line. Microglial cells were allowed to internalize IgG*-*opsonized latex beads for 40 min followed by an incubation with a secondary antibody Alexa Fluor 594 to distinguish extracellular and/or adherent beads (red labeling) from internalized beads (no fluorescent labeling) (Fig. 1a). Phagocytosis was addressed by counting the total number of internalized beads per cell. We showed that IgG bead phagocytosis increased with escalating concentrations of histamine, reaching about a 2.5-fold increase when 100 μM histamine was added to cells (*P* < 0.001, Fig. 1a, b). At this concentration, histamine did not interfere with cell death or proliferation (data not shown). Based on these results and on prior studies reported by us [8, 38, 39], we then used 100 μM histamine in further experiments, a concentration of pathophysiological relevance. In order to identify which histamine receptor was involved in phagocytosis, we treated microglial cells with the antagonists for each receptor before histamine treatment. Our results showed that only H1R antagonist (mepyramine maleate, 1 μM) significantly abolished histamine-induced phagocytosis when compared with histamine per se (*P* < 0.001, Fig. 1b). Consistently, an H1R agonist (2-pyridylethylamine, 100 μM) was able to mimic the effect induced by histamine (Fig. 1b). Antagonists for H2R (cimetidine, 5 μM), H3R (carcinine ditrifluoroacetate, 5 μM) or H4R (JNJ7777120, 5 μM) were not efficient in abolishing histamine-induced phagocytosis (Fig. 1b). These data suggest that histamine-induced FcγR-microglial phagocytosis of opsonized beads via H1R activation.

**Fig. 2:**
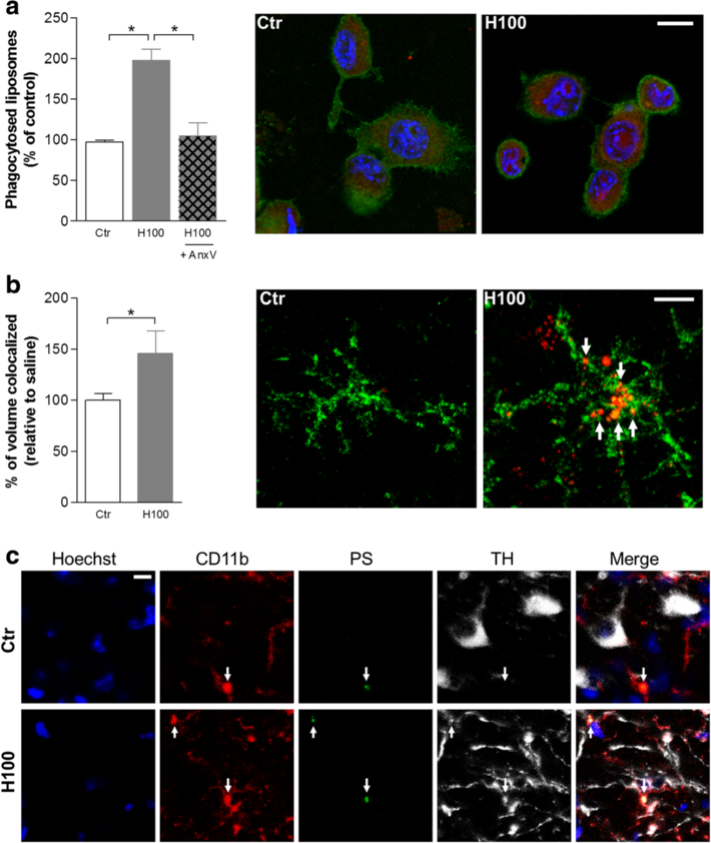
Histamine-induced microglial phagocytosis of PS particles/residues. **a** Bar graph displays the stimulatory effect of 100 μM histamine (H100) on the phagocytosis of phosphatidylserine (PS)-conjugated liposomes in vitro. Specific effect on PS-induced phagocytosis was ruled out by using annexin V (AnxV). Data are expressed as mean ± SEM (*n* = 2–10) and as a percentage of control. *P < 0.05, using one-way ANOVA followed by Bonferroni’s multiple comparison test. *Right panel*: representative photomicrographs illustrate the stimulatory effect of 100 μM histamine on microglial phagocytosis of PS liposomes in vitro. PS liposomes: *red labeling*; CD11b: *green labeling*; nuclei (Hoechst 33342): *blue. Scale bar* 10 μm. **b** The bar graph represents the volume of CD11b+ cells containing PS liposomes in SN slices from mice injected intracranially with 100 μM histamine for 18 h. Data are expressed as mean ± SEM (*n* = 4–6 mice) and as a percentage of saline. *P < 0.05, using an unpaired *t* test as compared with saline mice. *Right panel*: representative photomicrographs illustrate the stimulatory effect of histamine on microglial phagocytosis of PS liposomes in vivo. PS liposomes: *red labeling*; CD11b: *green labeling*; nuclei (Hoechst 33342): *blue. Arrows* highlight co-labeling events. *Scale bar* 10 μm. **c** Representative confocal photomicrographs showing that the stereotaxic injection with 100 μM histamine (H100) in the SN of adult mice for 3 days induced co-localization (highlighted with *white arrows*) between PS residues (*green*), CD11b labeling (*red*), and TH dopaminergic neuronal staining (*white*). This co-localization was less evident in saline animals. Cell nuclei were stained with Hoechst 33342 (in *blue*). *Scale bar* 10 μm

Then, we determined whether histamine could also modulate PSR-mediated phagocytosis using fluorescent PS or PC liposomes, as described in [33]. For this purpose, N9 microglial cells were exposed to 100 μM histamine for 6 h followed by 2 h incubation with PS or PC liposomes (Fig. 2a). We found that histamine significantly increased by twofold the phagocytosis/uptake of PS liposomes (*P* < 0.05; Fig. 2a). Annexin V which binds to PS residues was used as an inhibitor of PS-induced phagocytosis. As expected, annexin V fully abolished the histamine-induced uptake of PS liposomes (Fig. 2a). No significant differences were found in the levels of PC liposomes phagocytosis when cells were treated with histamine as compared to the controls (histamine: 139.7 ± 29.2 as compared with the control — set to 100 %, *n* = 3–4). Then, we evaluated the effect of histamine in microglia phagocytosis in vivo by injecting histamine in the SN of mice followed by PS liposomes. The volume of CD11b-positive (+) cells (resident microglial cells/invading macrophages) containing PS liposomes was quantified as described previously by others [33]. As expected, histamine significantly increased the percentage of volume of CD11b+ cells containing PS liposomes as compared with saline mice (*P* < 0.05; Fig. 2b). The physiological relevance of the aforementioned results was then validated by investigating the ability of microglial cells to phagocyte PS residues on the membrane of stressed/dying neurons upon histamine exposure in vivo. The stereotaxic injection of histamine in the SN for 3 days triggered a relative higher number of co-localization events between CD11b+, TH+, and annexin V+. These co-localization events were less evident in saline animals (Fig. 2c). Altogether, these results suggest that histamine induces phagocytosis of particles and cells displaying PS residues on their surfaces both in vivo and in vitro, likely increasing the vulnerability of stress/dying neurons to microglia phagocytosis.

### Histamine triggers microglial cytoskeleton modifications

**Fig. 3:**
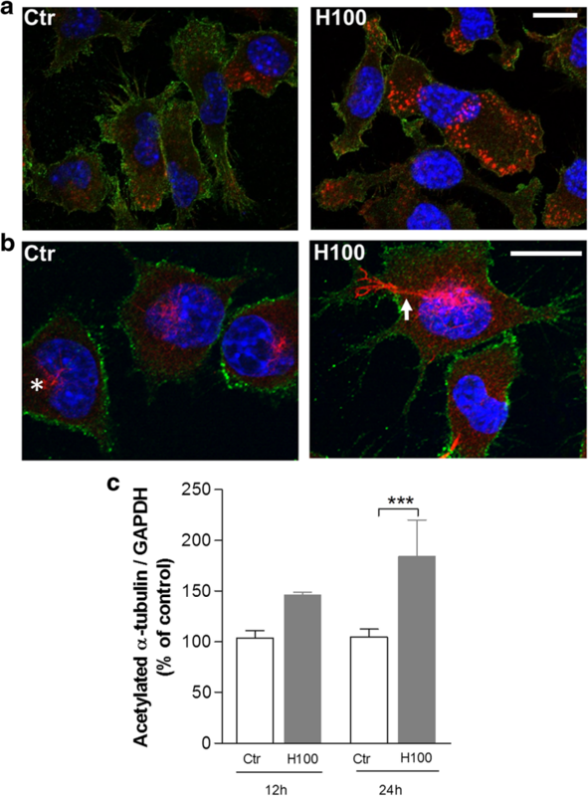
Histamine-induced microglial cytoskeleton rearrangement. **a** Representative confocal photomicrographs showing that 100 μM histamine (H100) induced membrane ruffling. Cells were stained for phalloidin (*red*), CD11b (*green*), and Hoechst 33342 (nuclei in *blue*). **b** Histamine also increased α-tubulin acetylation expression levels especially in some microglial processes (*white arrows*) as compared with the faint staining present in microtubule organization centers (*asterisk*) found in resting microglial cells, as detected by immunolabeling for acetylated α-tubulin (*red*), CD11b (*green*), and Hoechst 33342 (nuclei in *blue*). *Scale bars* 10 μm. **c** Bar graph displays the increased expression levels of acetylated α-tubulin in histamine-activated cells. Data are expressed as mean ± SEM (*n* = 4–8) and as a percentage of control. ***P < 0.001, using one-way ANOVA followed by Bonferroni’s multiple comparison test

To further explore the cytoskeleton modifications behind histamine-mediated phagocytosis, microglial cells were stimulated with 100 μM histamine for 1 h for actin filaments (phalloidin staining), and for 12 or 24 h for microtubule stabilization evaluation (acetylated α-tubulin protein levels). Histamine-induced membrane ruffling by actin polymerization and punctuate staining in structures involved in the initiation of phagocytosis (Fig. 3a). In addition, we found that in unstimulated microglial cells (control) acetylated α-tubulin staining was found predominantly confined to the centrosome tubules (Fig. 3b). In contrast, histamine induced an increase of acetylated α-tubulin labeling particularly in several microglial processes that may be involved in the stabilization of phagocytic cups/protrusions (Fig. 3b). In accordance, acetylated α-tubulin protein expression levels were significantly increased by histamine (1.8-fold increase, *P* < 0.001) at 24 h post-treatment as compared with the controls (Fig. 3c). These results suggest that histamine induces cytoskeleton alterations associated with microglia phagocytosis dynamics.

### Oxidative stress contribution to histamine-induced microglial phagocytosis

**Fig. 4:**
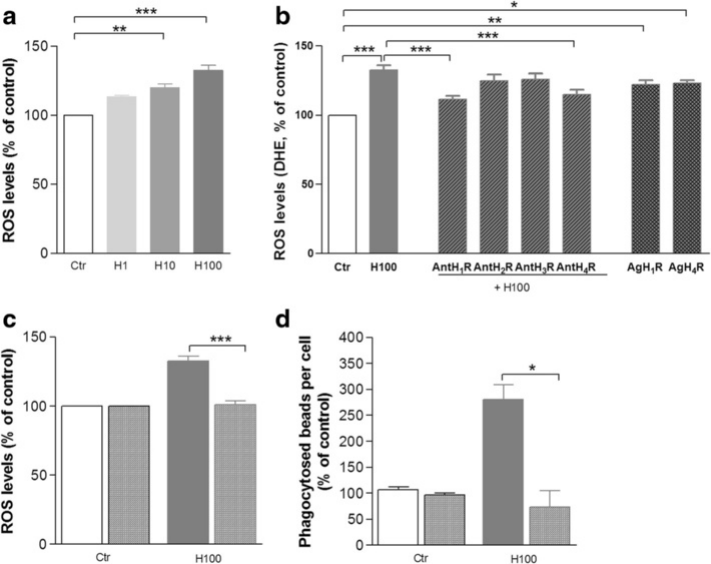
Histamine-induced ROS generation contributes to microglial phagocytosis. **a** Only 10 (H10) and 100 μM histamine (H100) significantly increased the levels of ROS as compared with untreated controls (Ctr). **b** Cellular ROS production induced by 100 μM histamine was blocked by an H1R antagonist (AntH1R, mepyramine maleate, 1 μM) or H4R antagonist (AntH4R, JNJ7777120, 5 μM) and mimicked by an H1R agonist (AgH1R, 4-methylhistamine dihydrochloride, 20 μM) or a H4R agonist (4-methylhistamine dihydrochloride, 20 μM). The involvement of other receptors was excluded since the application of their respective antagonists did not interfere with ROS levels induced by histamine (H2R antagonist (cimetidine), 5 μM; H3R antagonist (carcinine ditrifluoroacetate), 5 μM). Apocynin (5 μM) was used to rule out the involvement of the NADPH oxidase system in cellular production of ROS (**c**) and in the phagocytosis of IgG latex beads (**d**) induced by histamine. Apocynin-treated cells are represented in *slashed columns* both in (**c** and **d**). Data are expressed as mean ± SEM (*n* = 3–16) and as a percentage of control. *P < 0.05, **P < 0.01, and ***P < 0.001, using one-way ANOVA followed by Bonferroni’s multiple comparison test

**Fig. 5:**
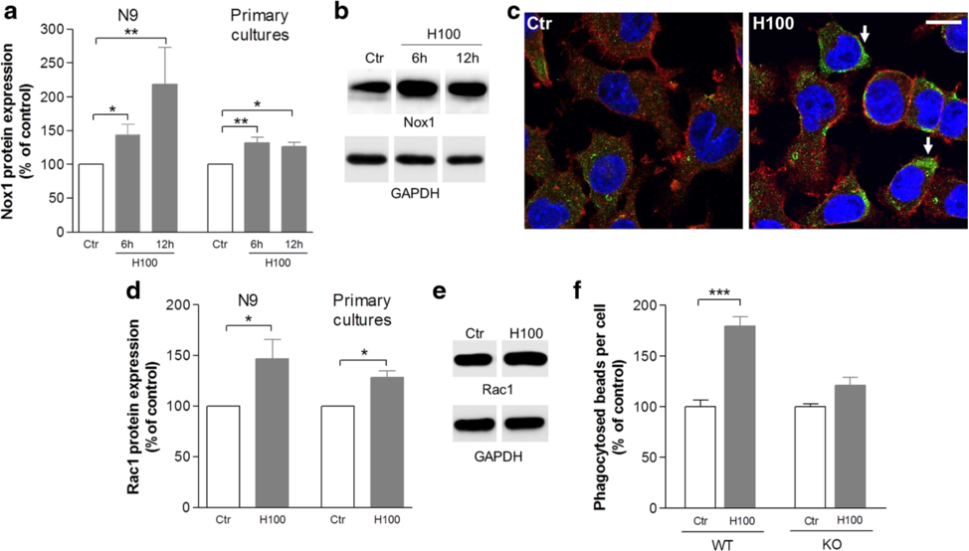
Rac-1/Nox1 signaling is involved in microglial phagocytosis triggered by histamine. **a** Bar graph depicting Nox1 protein expression levels upon treatment with 100 μM histamine (H100) for 6 or 12 h, both in the N9 cell line and primary microglial cell cultures. Data are expressed as mean ± SEM (*n* = 4–10) and as a percentage of control. *P < 0.05 and **P < 0.01, using Bonferroni’s multiple comparison test as compared with control. **b** Representative Nox1 (65 kDa) and GAPDH (37 kDa) Western blots in primary microglial cell cultures. **c** Representative confocal photomicrographs showing that 100 μM histamine-induced Nox1 expression in the luminal side of the plasma membrane of N9 microglial cells (highlighted by *white arrows*). Cells were stained for CD11b (*red*), Nox1 (*green*), and Hoechst 33342 (nuclei in *blue*). *Scale bar* 10 μm. **d** Bar graph depicting Rac1 protein expression levels upon treatment with 100 μM histamine (H100) for 1 h, both in the N9 cell line and primary microglial cell cultures. Data are expressed as mean ± SEM (*n* = 3–8) and as a percentage of control. *P < 0.05 using paired *t* test as compared with control. **e** Representative Rac1 (22 kDa) and GAPDH (37 kDa) Western blots in primary microglial cell cultures. **f** Bar graph displays the effect of histamine on the phagocytosis of IgG latex beads in Nox1 knockout mice (KO) and their respective wild-type (WT) littermates. Data are expressed as mean ± SEM (*n* = 4) and as a percentage of control. ***P < 0.001 using Bonferroni’s multiple comparison test as compared with control

Then, we evaluated the effect of histamine on ROS production and the putative contribution of ROS for microglial phagocytosis. As shown in Fig. 4a (N9 cell line) and in Additional file 1: Figure S1 (primary murine microglia cells), 100 μM histamine significantly increased ROS levels when compared to control (*P* < 0.001). ROS production induced by 100 μM histamine was fully inhibited by H1R or H4R antagonists (Fig. 4b and Additional file 1: Figure S1) and mimicked by H1R (*P* < 0.01) or H4R (*P* < 0.05) agonists (Fig. 4b). The co-administration of both H1R and H4R antagonists with histamine blocked histamine-induced ROS production to the same level as both antagonists individually (data not shown). These data suggest that histamine-induced ROS production occurs via H1R and H4R activation, both in N9 cell line and primary murine microglia cell cultures. Then, apocynin was used to clarify the specific role of the Nox system in microglia ROS generation induced by histamine. Apocynin significantly inhibited histamine-induced ROS production in N9 microglial cells to levels similar to the controls (*P* < 0.001, Fig. 4c). Interestingly, apocynin also fully abrogated histamine-induced IgG beads phagocytosis (*P* < 0.05, Fig. 4d). The concentration of apocynin used did not interfere with microglial cell death as evaluated by terminal deoxynucleotidyl transferase dUTP nick end labeling (TUNEL) and propidium iodide (PI) assays (data not shown). These results suggest that Nox activation and subsequent ROS production are involved in histamine-induced phagocytosis. Accordingly, histamine significantly increased Nox1 protein levels at 6 and 12 h post-treatment, both in the N9 cell line and primary murine microglial cells (Fig. 5a, b). Nox1 labeling was found preferentially at the luminal side of the plasma membrane, suggesting membrane recruitment of Nox1 signaling cascade (Fig. 5c). In contrast, no differences were observed regarding Nox2 and Nox4 expression levels in histamine-treated cells as compared with the controls (Additional file 2: Figure S2). Functional activation of Nox1 is dependent on the activation of a small GTPase, Rac1. We found that histamine increased the expression levels of Rac1 1 h after treatment, both in the N9 cell line and primary murine microglial cells (Fig. 5d, e). To prove the involvement of Nox1 signaling pathway on histamine-induced phagocytosis, we performed the same IgG bead phagocytosis protocol on primary cultures of microglial cells obtained from Nox1-KO mice and matched WT littermates. We found that Nox1 deficiency inhibits histamine-induced phagocytosis (Fig. 5f). Altogether, these results suggest that the Nox1/Rac1 signaling and the resultant ROS production are involved in histamine-induced microglial phagocytosis.

### Histamine-induced microglial activation modulates DA neuronal survival via H1R activation

**Fig. 6:**
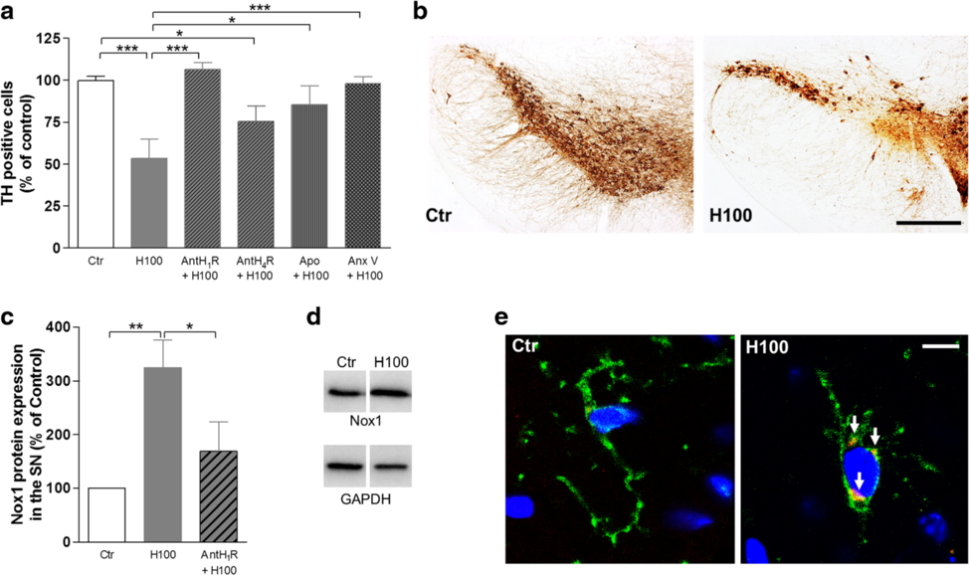
Histamine induces DA toxicity via H1R activation and involves oxidative stress and PS-mediated phagocytosis. **a** Bar graph depicts the percentage of TH+ cells in the SN of mice. A significant reduction in the number of TH+ neurons was found in 100 μM histamine (H100)-treated mice as compared with saline. Antagonist (Ant) for H1R abolished the toxicity induced by histamine. The toxic effect induced by histamine involves the production of ROS and PS-induced phagocytosis, since both apocynin (“Apo”, NADPH oxidase inhibitor) and annexin V (“AnxV”, blocker of PS residues) could inhibit dopaminergic toxicity. Data are expressed as mean ± SEM (*n* = 4–7 mice) and as a percentage of saline animals. *P < 0.05 and ***P < 0.001, using one-way ANOVA followed by Bonferroni’s multiple comparison test. **b** Representative immunostainings for TH in the SN of adult mice. A notable decrease in the number of TH+ cells could be observed in mice injected with histamine when compared with saline animals. **c** Bar graph depicts Nox1 protein expression levels in the SN. Increased expression of Nox1 protein found in histamine (H100)-treated mice was completely abolished by the H1R antagonist. Data are expressed as mean ± SEM (*n* = 4 mice). *P < 0.05 and **P < 0.01, using one-way ANOVA followed by Bonferroni’s multiple comparison test. **d** Representative Nox1 (65 kDa) and GAPDH (37 kDa) Western blots in the SN of adult mice in vivo. **e** Representative confocal photomicrographs showing that histamine-induced Nox1 expression in the perinuclear region of microglial cells in the SN in vivo. Cells were stained for CD11b (*green*), Nox1 (*red*), and Hoechst 33342 (nuclei in *blue*). *White arrows* highlight Nox1 staining in microglial cells. *Scale bar* 10 μm

We then explored the consequences of histamine-induced microglia activation in DA neuronal survival in vivo. We found that the stereotaxic injection of histamine in the SN of adult C57BL/6 mice for 7 days induced about 40–50 % decrease of TH+ cells as compared with saline-treated mice (*P* < 0.001; Fig. 6a, b). To disclose the involvement of histamine receptors, we then co-injected histamine with the H1R or H4R antagonists in the SN. We found that only H1R antagonist was able to abolish the neurotoxic effect of histamine (Fig. 6a). Histamine-induced DA toxicity was also abolished by annexin V and apocynin (AnX V + H100: 108.9 ± 3.0; Apo + H100: 84.9 ± 10.9; *n* = 5, as compared with saline mice — set to 100 %; Fig. 6a). Annexin V or apocynin per se did not modify significantly the number of TH+ cells as compared with saline animals (data not shown). In accordance with previous in vitro data (Fig. 5), Nox1 protein levels were increased in the SN of histamine-treated mice (Fig. 6c, d). H1R antagonist completely abolished histamine-induced Nox1 expression (Fig. 6c). Nox1 labeling was found predominantly in the perinuclear region of microglial cells (Fig. 6e). Altogether, our findings suggest that histamine, via H1R activation, induces microglial activation and ultimately the death of susceptible DA neurons, possibly by Nox1-dependent oxidative signaling and PS-mediated microglial phagocytosis.

## Discussion

Herein, we aimed to disclose the role of histamine and its receptors in microglia activation, namely in phagocytosis and ROS production, and ultimately to explore the functional consequences of this inflammatory response in DA neuronal survival.

First, we found that histamine induces the phagocytosis of IgG-opsonized latex beads via H1R activation. This is in accordance with other reports showing that histamine can also induce phagocytosis in macrophages [40, 41]. In contrast, other reports argue that histamine inhibits macrophage phagocytosis [42, 43]. These contradictory studies may be due to the different types of cells used, range of histamine concentrations, and/or different experimental protocols. On the other hand, microglial cells have other surface receptors that recognize PS residues exposed on the surface of cells that underwent apoptosis or were subjected to certain stressing agents. The PS exposure acts as “eat-me” signals that can be recognized by microglial cells as targets to be eliminated [44, 45]. We found that histamine could also potentiate phagocytosis/uptake of PS liposomes. Annexin V was able to inhibit histamine-induced PS phagocytosis, demonstrating that this process depends on the exposure of PS residues by liposomes. Interestingly, histamine induces both types of FcγR- and PSR-induced microglia phagocytosis, apparently triggering opposite inflammatory responses to the engulfment of distinct cargo. Classically, FcγR phagocytosis is associated to a M1 pro-inflammatory microglial phenotype, whereas M2 anti-inflammatory phenotype is mainly associated to PSR-phagocytosis. However, classic activation paradigms driving an M1 phenotype also lead to enhanced engulfment of apoptotic cells in vitro [46]. On the other hand, M2 phenotype driven by IL-4 and IL-13 increases the phagocytosis of myelin [47], whereas a M2 phenotype induced by glioma cancer stem cells reduces phagocytosis [48]. This suggests that histamine may be a versatile immune modulator by activating a particular phagocytosis pathway depending on a particular context.

Activated microglia also release an inflammatory cocktail, including cytokines, phagocytic adaptor proteins (e.g., Milk Fat Globule-EGF Factor 8 Protein - MFG-E8), ROS, and NO, that may interfere with neuronal survival. Previously, we showed that histamine induces NO release by microglial cells [8]. Neither IL-1β nor tumor necrosis factor (TNF)-α was released upon histamine stimulation in the N9 microglial cell line that we used [7]. Herein, we show that histamine induces ROS production via H1R and H4R activation. A recent study showed that histamine induces TNF-α, IL-6 and ROS production and loss of mitochondrial membrane potential in rat primary microglial cultures, primarily via H1R and H4R activation [10]. Both H1R and H4R activate phospholipase C (PLC) [49, 50]. PLC can activate either PLC-inositol triphosphate (IP3) leading to intracellular calcium increase or PLC-protein kinase C (PKC) which in turn initiates nuclear factor (NF)-kappaB activation and can induce increased gene transcription, including inducible nitric oxide synthase (iNOS) expression and NO release [51–53]. This redundant and superimposed signaling pathways induced by H1R and H4R may mediate the release/production of inflammatory mediators (including NO and ROS) differently involved in several steps of microglial activity. Herein, our results demonstrated that histamine may also induce Rac1/Nox1 signaling triggering increased ROS levels which are responsible for microglia phagocytosis. Previous reports described that in microglia [54] and in DA neurons [55], Nox1 transcriptional regulation is modulated by PKCdelta. As mentioned above, this is especially important taking into account that H1R may activate PLC which is known to be the upstream regulator of PKCdelta activity. Moreover, it was recently reported that in dorsal root ganglion neurons, histamine activates PKCdelta via H1R [53]. Based on these assumptions, we may speculate that the downstream histamine signaling pathway may involve the activation of PLC by H1R, followed by PKCdelta activation inducing increased Nox1 expression and activation culminating in increased ROS production. In a number of studies, the involvement of Nox2 in microglia-dependent DA neurotoxicity has been also observed [56, 57]. These findings raise the question on whether other Nox isoforms are involved in microglia inducing oxidative stress in the nigrostriatal DA pathway. In the present work, we observed that Nox1 has a critical role in microglia phagocytosis as shown by experiments using Nox1-KO microglia cultures. Nevertheless, it is also plausible that microglia Nox2 may have a participation, probably in an upstream step, that with time is abolished given place to Nox1-inducing oxidative stress by microglia. In the present work, we are also showing that histamine increases PS-mediated microglia phagocytosis. Oxidation of PS is crucial for its externalization, and it was previously reported that Nox-dependent ROS generation is responsible for the selective oxidation of PS in neutrophils [58]. In addition, Brown and Neher [44] have shown that peroxynitrite produced by microglia is responsible for the externalization of PS on target cells to potentiate its phagocytosis. Also in this line, a study has shown that in activated microglia the O^2−^ produced by Nox1 may increase the levels of peroxynitrite that may diffuse and affect neighboring target cells [19]. Altogether, this supports the outcome of our results showing that in microglia, histamine increase Nox1 expression which is responsible for increasing ROS production and PS oxidation in target cells potentiating its phagocytosis.

The downstream signaling pathways induced by both FcγR and PSR receptor activation lead to the remodeling of the microglial cytoskeleton through actin polymerization which is involved in the formation of pseudopodia. The formation of pseudopods is coincident with local remodeling of the cortical actin cytoskeleton and the formation of phagocytic cups involved in target engulfment [16, 59]. In fact, we observed that histamine induced a robust actin punctuate staining in membrane ruffles. In accordance, we previously showed that LPS promotes microglial phagocytosis by modulating actin cytoskeleton reshuffling and the formation of phagocytic cups [60]. We also observed that histamine induced an increase of acetylated α-tubulin levels, localized preferentially along microglial processes. Acetylation of α-tubulin is a post-translational modification that serves as a marker of microtubule stabilization which is essential for spreading and phagocytosis [61]. These cytoskeleton modification events involving both actin polymerization and tubulin stabilization require activation of Rho GTPases, namely Rac1 and cdc42 [62–64]. This is in agreement with the increased levels of Rac1 expression induced by histamine in our microglial cell line. Rac1 is involved in the activation of Nox, in particular the Nox1 isoform, but also in cytoskeleton alterations required for microglial phagocytosis.

We then hypothesize that histamine-induced microglial activation could be related to the modulation of DA survival in vivo. In accordance with previous studies [27, 65], histamine was able to induce DA neuronal death accompanied by microglial reactivity. The neurotoxic effect induced by histamine was prevented by blocking H1R. Recently, it was also shown that histamine impairs midbrain DA development in vivo via H1R activation [66]. However, so far, the cellular and molecular mechanisms behind histamine-induced DA toxicity in the adult brain in vivo are not known. We hypothesize that histamine may induce an inflammatory milieu that encompasses increased ROS levels that triggers DA neurons to expose PS on their outer membrane leaflet stimulating microglial phagocytosis, oxidative stress, and ultimately DA neuronal death. Indeed, we found that histamine induces DA neuronal death via H1R activation, an effect accompanied with increased Nox1 protein levels. Interestingly, in the present experimental in vivo paradigm, Nox1 was found in the perinuclear region of the microglial cells. Additionally, Nox1 expression was also found in neurons (Additional file 3: Figure S3), as we have previously showed that Nox1 is upregulated on neurons in response to other in vivo injury paradigms such as ischemia or paraquat and 6-hydroxydopamine (6-OHDA)-mediated neuronal death [34, 67, 68]. Therefore, we cannot exclude the putative involvement of neuronal Nox1 activation in the modulation of DA cell survival in vivo. We also found that histamine-induced PS exposure in the membranes of DA neurons and annexin V was able to abolished histamine-induced DA cell death. We suggest that oxidative stress induced by histamine, such as the production of peroxynitrite formed by the reaction between NO and O^2−^, may trigger PS exposure on DA neurons which may become vulnerable to microglial phagocytosis [14, 45]. This particular type of neuronal loss referred as phagoptosis was also observed in animal models of PD and could be prevented by using phagocytosis inhibitors [69]. In fact, the "eat-me" signaling induced by PS exposure was reversible, so that blocking of phagocytosis through binding of PS by annexin V was sufficient to rescue DA toxicity induced by histamine. Recent studies also showed that microglial cells appear to migrate towards intoxicated/damaged DA neurons and present clear phagocytic characteristics, such as engulfing gliaptic contacts, nucleation/clustering of the actin cytoskeleton, and an increase in Golgi apparatus protein machinery in the SN of both 1-methyl-4-phenyl-1,2,3,6-tetrahydropyridine (MPTP)-treated mice and macaques [70, 71]. Gliaptic contacts include the contact of microglial cell processes and cell bodies with DA neurites and cell bodies. Importantly, the inhibition of Rho-associated kinase (ROCK) activity prevents microglial migration and the formation of gliapses to protect damaged DA neurons from elimination. Altogether, both phagoptosis and the formation of gliaptic contacts may be involved in the unnecessary phagocytic elimination of DA neurons. Thus, blocking microglial migration and phagocytic activity might preserve degenerating neurons and induce neuroprotection. However, we cannot exclude that higher histamine concentrations may induce direct DA neuronal death without involving “primary phagocytosis” by microglia. In fact, we have previously shown that the secretome of histamine-treated microglia cells trigger DA cell death in vitro [8], and the direct application of histamine to a N27 rat DA cell line was able to induce a slight reduction of cell viability (about 25 % reduction, data not show). This may suggest that histamine induces DA cell loss by both soluble and contact factors, and the contribution of each component may depend on the complexity of the experimental model used (in vivo versus in vitro) and the interaction with other brain cells. Therefore, molecules that prevent neuroinflammation and microglia activation, in particular microglial phagocytosis, may offer prospective clinical therapeutic benefit for neurodegenerative disease associated with an inflammatory profile, such as PD. Yet, future studies are needed to further disclose the role of histamine and antihistamines in PD, by using toxin-induced (e.g., MPTP or 6-OHDA) models combined with H1R conditional KO models to discriminate the temporal and differential contribution of H1R in both dopaminergic neurons and microglia. In accordance with our data, others also showed a neuroprotective and/or anti-inflammatory effect of antihistamines (e.g., H1R or H3R antagonists) in vivo in models of amyotrophic lateral sclerosis [72], temporal lobe epilepsy [73], and ischemia [74]. In this context, histamine receptors, especially microglia H1R, may be an interesting target to induce neuroprotection in neuroinflammatory-related diseases.

## Conclusions

In the healthy brain, histamine is present at nanomolar concentrations in the CSF of humans and rodents [75]. Importantly, circulating levels of histamine and histaminergic innervations are robustly increased following brain injury or degeneration. Under these pathological conditions, the inflammatory response may trigger degranulation of mast cells, leading to a massive release of histamine in the CSF and in the brain parenchyma [22, 23, 76]. These evidences support the pathophysiological relevance of the histamine concentrations used in this study. Therefore, unraveling the cellular and molecular mechanisms triggered by histamine in neurodegenerative conditions, and particularly in PD, is of outmost relevance. In particular, the role of histamine on microglial activity has been recently uncovered and we were the first to show that N9 microglial cells express all histamine receptors and that histamine modulates microglial motility and cytokine release [7]. Herein, we show that histamine is an important modulator of microglial phagocytosis and ROS production, both components involved in the vulnerability and cell death of DA neurons in the SN in vivo. To the best of our knowledge, we were the first to show that the Nox signaling is involved in histamine-induced neuroinflammation and neurodegeneration. Importantly, histamine H1 antihistamines may be used as efficient therapeutic targets to prevent histamine-induced microglia activation and subsequent DA neuronal death in the context of PD and, eventually, other neurodegenerative diseases which are accompanied by microglia inflammation.

## Abbreviations

DA, dopaminergic neurons; HR, histamine receptor; IL, interleukin; NADPH, nicotinamide adenine dinucleotide phosphate; NO, nitric oxide; Nox, NADPH oxidase; PD, Parkinson’s disease; PS, phosphatidylserine; ROS, reactive oxygen species; SN, substantia nigra

## Additional files

Additional file 1: Figure S1. Histamine-induced ROS production via H1R and H4R activation in primary microglial cell cultures. Cellular ROS production induced by 100 μM histamine (H100) was blocked by an H1R antagonist (AntH1R, mepyramine maleate, 1 μM) or H4R antagonist (AntH4R, JNJ7777120, 5 μM). Data are expressed as mean ± SEM (*n* = 5) and as percentage of control. ***P* < 0.01 and ****P* < 0.001, using one-way ANOVA followed by Dunnett’s test. (TIF 251 KB) Additional file 2: Figure S2. Nox2 and Nox4 expression. Bar graphs depict Nox2 (A) and Nox4 (B) protein expression levels upon treatment of microglial cells with 100 μM histamine for several time points. LPS (100 ng/ml) was used as a positive control. Data are expressed as mean ± SEM (*n* = 2–8) and as a percentage of control. **P* < 0.05, one-way ANOVA, Bonferroni’s multiple comparison test. (TIF 161 KB) Additional file 3: Figure S3. Histamine-induced Nox1 immunostaining in dopaminergic neurons in the SN in vivo. Representative confocal photomicrographs showing that histamine-induced Nox1 expression in the dopaminergic cells in the SN in vivo. Cells were stained for TH (green), Nox1 (red), and Hoechst 33342 (nuclei in blue). Scale bar 10 μm. (TIF 98 KB)
