# Supplementary figures and images for: Histamine induces microglia activation and dopaminergic neuronal toxicity via H1 receptor activation
Source: J Neuroinflammation. 2016 Jun 4;13:137. doi: 10.1186/s12974-016-0600-0 (PMC4893260; doi:10.1186/s12974-016-0600-0)

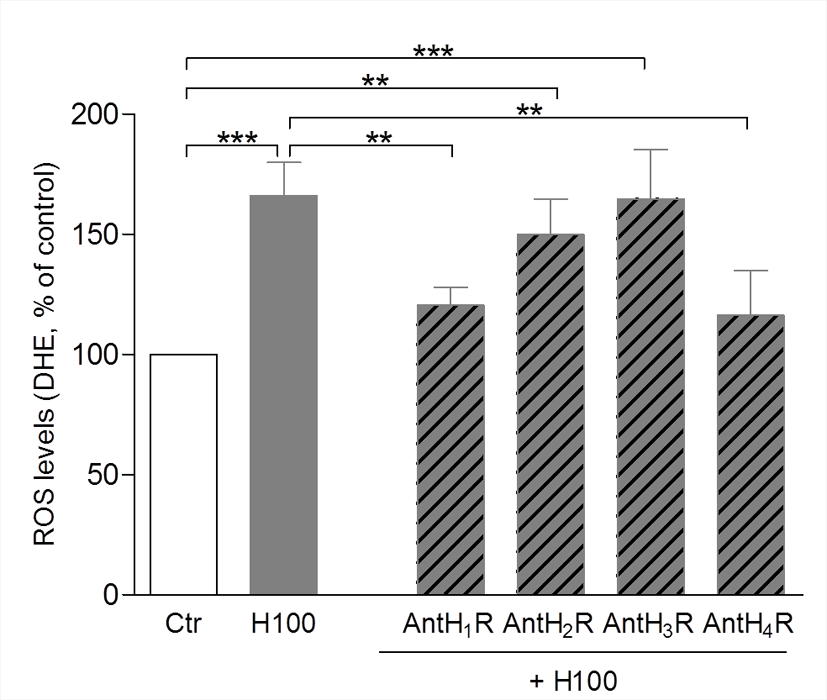

Supplement: Additional file 1: Figure S1. — Histamine-induced ROS production via H1R and H4R activation in primary microglial cell cultures. Cellular ROS production induced by 100 μM histamine (H100) was blocked by an H1R antagonist (AntH1R, mepyramine maleate, 1 μM) or H4R antagonist (AntH4R, JNJ7777120, 5 μM). Data are expressed as mean ± SEM (n = 5) and as percentage of control. **P < 0.01 and ***P < 0.001, using one-way ANOVA followed by Dunnett’s test. (TIF 251 KB) [file 12974_2016_600_MOESM1_ESM.tif]

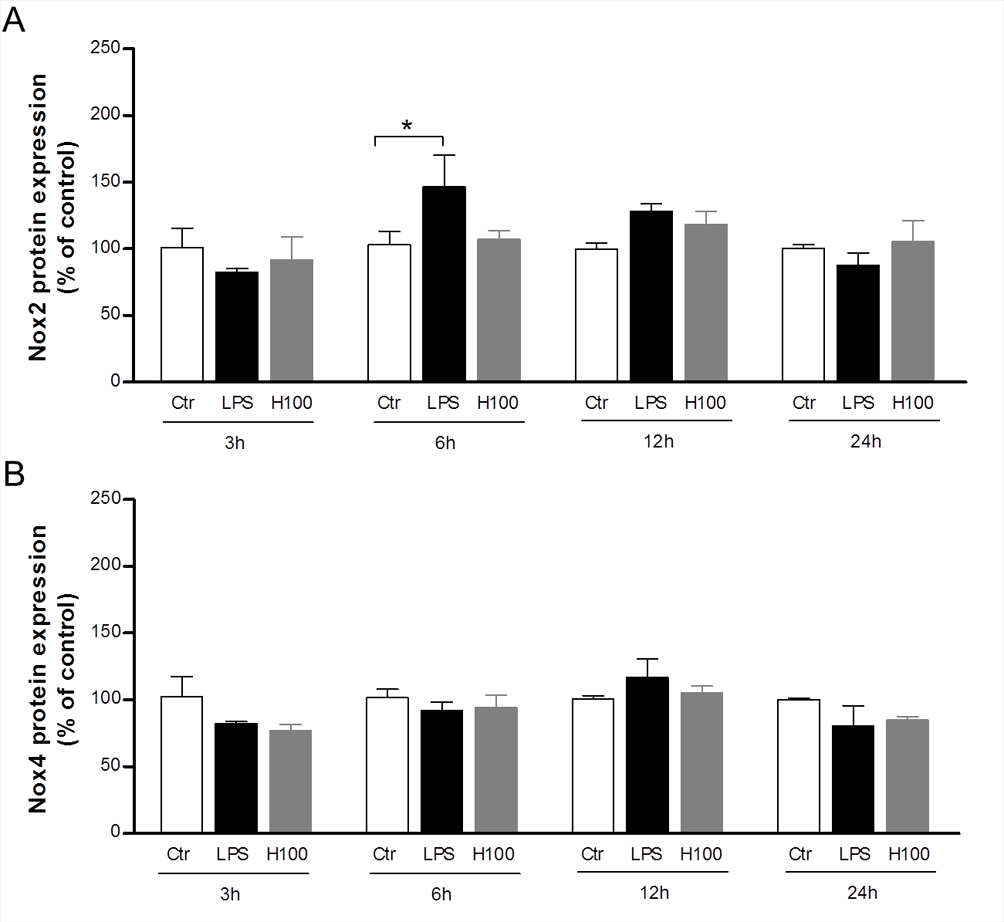

Supplement: Additional file 2: Figure S2. — Nox2 and Nox4 expression. Bar graphs depict Nox2 (A) and Nox4 (B) protein expression levels upon treatment of microglial cells with 100 μM histamine for several time points. LPS (100 ng/ml) was used as a positive control. Data are expressed as mean ± SEM (n = 2–8) and as a percentage of control. *P < 0.05, one-way ANOVA, Bonferroni’s multiple comparison test. (TIF 161 KB) [file 12974_2016_600_MOESM2_ESM.tif]

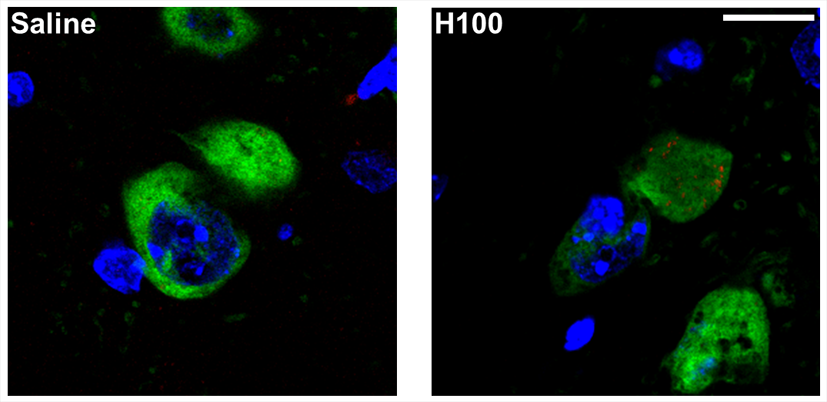

Supplement: Additional file 3: Figure S3. — Histamine-induced Nox1 immunostaining in dopaminergic neurons in the SN in vivo. Representative confocal photomicrographs showing that histamine-induced Nox1 expression in the dopaminergic cells in the SN in vivo. Cells were stained for TH (green), Nox1 (red), and Hoechst 33342 (nuclei in blue). Scale bar 10 μm. (TIF 98 KB) [file 12974_2016_600_MOESM3_ESM.tif]
